# AVATAR therapy: results of a phase 2/3 Clinical Trial of a blended digital therapy for auditory verbal hallucinations

**DOI:** 10.1192/j.eurpsy.2025.52

**Published:** 2025-08-26

**Authors:** T. K. J. Craig

**Affiliations:** Institute of Psychiatry, Psychology and Neuroscience, King’s College London, London, United Kingdom

## Abstract

**Abstract:**

Avatar therapy is a digital therapy supported by a therapist in which voice hearers dialogue with a digital embodiment of their most distressing voice. Our previous randomised controlled trial of AVATAR therapy showed 6 sessions had a superior and substantial reduction in the severity of voices compared to that achieved by supportive counselling. Limitations included that the study was carried out in a single site, and six sessions limited what might be achieved by an extended approach to include a greater focus on individual biographical characteristics that had a bearing on the voice hearing experience . In the latest AVATR2 trial, we aimed to replicate these early results and deliver therapy by a wider workforce across several centres in the UK. We also tested the effectiveness of two forms of therapy -AVATAR Brief 6 sessions (AV-BRF) and AVATAR-Extended 12 sessions (AV-EXT). In this presentation I report the results of this clinical trial. We hypothesised that both forms of therapy alongside treatment as usual would be effective and superior to treatment as usual alone in reducing voice related distress (the primary outcome) at 16 and 28 weeks follow up. We found that both treatmetns did indeed reduce distress at 16 weeks and that this reduction was maintained at 28 weeks though was no longer statistically significant in compoariuson to treatment as usual at this point. The frequency of voices also reduced at both time points for AV-EXT that also achieved several other important outcomes in cluding enhanced wellbeing and reductions in delusional beliefs associated with the voice. There were no related serious adverse events. A health economic analysis was also carried out that also supports AV-EXT The next phase of the development of AVATAR therapy towards release for routine use is briefly described.

**Disclosure of Interest:**

T. K. J. Craig Grant / Research support from: Research Grants Wellcome Trust; NIHR UK

